# The effect of composite resin preheating on marginal adaptation of class II restorations

**DOI:** 10.4317/jced.56625

**Published:** 2020-07-01

**Authors:** Farideh Darabi, Reza Tayefeh-Davalloo, Seyedeh-Maryam Tavangar, Fereshteh Naser-Alavi, Mozhdeh Boorboo-Shirazi

**Affiliations:** 1 Associate professor, Department of Operative Dentistry, School of Dentistry, Guilan University of Medical Sciences, Rasht, Iran; 2 Assistant professor, Department of Operative Dentistry, School of Dentistry, Guilan University of Medical Sciences, Rasht, Iran; 3 Dentist, School of Dentistry, Guilan University of Medical Sciences, Rasht, Iran

## Abstract

**Background:**

One of the problems with a high filler content composite resins is gap formation at restorative material–tooth interface. The present study investigated the effect of preheating composite resins on the formation of marginal gap in Cl II restorations.

**Material and Methods:**

In this *in vitro* study Sixty Cl II cavities were prepared on the mesial and distal surfaces of 30 extracted premolar teeth. The gingival floor of cavities was placed 1 m below the CEJ. The samples were randomly allocated to 4 groups for restoration placement: group 1, Filtek P60 composite resin at room temperature; group 2, Filtek P60 composite resin at 68°C; group 3, X-tra fil composite resin at room temperature; and group 4, X-tra fil composite resin at 68°C. After a thermocycling procedure, the teeth were sectioned longitudinally in a buccolingual direction. Then the marginal gaps of the samples were measured at proximal and gingival margins under a scanning electron microscope at ×2000 magnification in µm. The data were analyzed with SPSS 21, using one-way ANOVA, post hoc Tukey tests and paired t-test (α=0.05).

**Results:**

Groups 2 and 4 exhibited significantly lower marginal gaps, compared to groups 1 and 3, at both enamel (*P*<0.0001 and *P*=0.001, respectively) and dentinal walls (*P*<0.0001). In all the groups, there was significantly less marginal gaps at composite-enamel wall compared to composite-dentin wall interfaces (*P*<0.0001). There was no significant difference between groups 1 and 3 and groups 2 and 4 in enamel walls (*p*= 0.96, *p*= 0.99 respectively) and dentinal walls (*p*= 0.85, *p*=0.98 respectively).

**Conclusions:**

Preheating resulted in a decrease in marginal gaps in both composite resins. The effect of composite resin type on marginal adaptation was the same.

** Key words:**Composite resin, dental marginal adaptation, preheating.

## Introduction

The frequency of posterior composite resin restorations is on the increase significantly due to the esthetic appearance and conservative nature of the material and advances in their physicomechanical properties. One of the most common problems associated with composite resin restorations is poor adaptation and formation of gaps between the restorative material and the tooth structure, resulting in some problems, including microleakage of oral fluids, postoperative sensitivity and recurrent caries (1). The clinical success of composite resin restorations dramatically depends on the properties of the material, including polymerization shrinkage, viscosity, packing capacity and bonding ability (2).

Although an increase in the filler content of commonly used high-viscosity composite resins results in an improvement in the physicomechanical and packing properties of these materials, it makes it challenging to adapt the restorative material to the cavity walls, leading to the formation of interfacial gaps and an increase in microleakage. One of the techniques suggested for solving the adaptation problems of composite resins is to use flowable composite resins as a liner before placing a composite resin with higher filler content in the cavity. However, this method increases the technique sensitivity of the procedure and decreases the durability of the restoration due to the lower filler content of flowable composite resin (3). Another technique to improve the marginal adaptation is the preheating a high-viscosity and packable composite resin up to 68°C before placing it in the cavity and light-curing it (4). It has been demonstrated that preheating composite resin decreases its viscosity and thickness, increasing its flow and adaptation with the cavity walls (5,6). In addition, preheating increases the polymerization rate and microhardness of composite resin, improving its physicomechanical properties (7). The effect of heat, due to a preheated composite resin, on the increase in pulpal temperature is minimal (approximately 2°C), which can be tolerated by the pulp. It should be pointed out that an increase in polymerization rate of composite resin might increase polymerization shrinkage and the stress resulting from it (8).

Recently, bulk-fill composite resins have been designed for placing the composite in bulk in the cavity. Changing the initiator in these composite resins making it possible to place them in layers measuring >4 mm in thickness, which decreases the time required for placing the material in the cavity (9). The main concern about the curing process of bulk-fill composite resins is the amount of polymerization shrinkage, polymerization stresses and the subsequent gap formation (10).

Since the flowability of composite resins is different in terms of the brand and the type of preheated composite resin, and composite resins exhibit different behaviors after heat treatment(6), wide variations are expected in the viscosity of composite resins after preheating, depending on the chemical structure of composite resin.

Considering the importance of the marginal adaptation of composite resin restorations, the existing variations in their types and structural differences in these materials and their possible effects on the behaviors of preheated composite resins, this study evaluated the effect of preheating of two types of packable composite resins, i.e., Filtek P60 (3M, ESPE,USA) and X-tra fil (VOCO, Germany) on gap formation at enamel and dentin margins of Cl II restoration using a scanning electron microscope (SEM).

## Material and Methods

The study protocol was approved by the Ethics Committee at Guilan University of Medical Science (IR.GUMS.REC.1395.125). Table 1 presents the characteristics of the materials used in the present study.

**Table 1:**
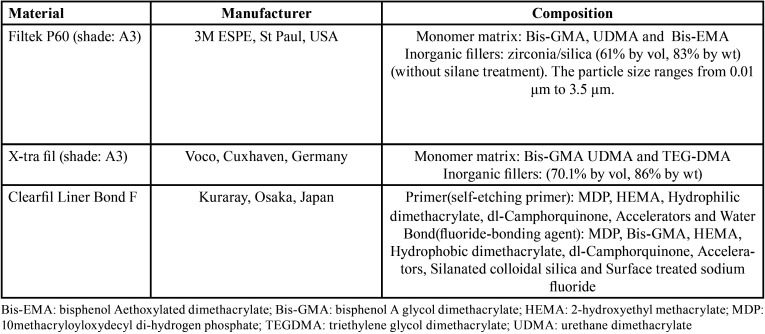
The materials used in the present study.

This *in vitro* study was carried out on 30 human premolar teeth extracted for orthodontic reasons. The teeth were sound, with no caries, cracks or anomalies as evaluated visually and under a stereomicroscope. All the teeth were cleaned with a scaling curette and stored for one week in 0.5% chloramine T solution, followed by storage in distilled water at refrigerator temperature until they were tested. Standard Cl II cavities in box form, measuring 3 mm buccolingually and 1.5 mm in the axial depth, were prepared, with butt joint margins, on the mesial and distal surfaces of the teeth. The gingival margins of all the cavities were placed 1 mm apical to the CEJ. The cavities were prepared with sharp diamond fissure burs (Stoddard, England), measuring 1 mm in diameter in a high-speed handpiece under water and air coolant. The burs were replaced by new ones after every five cavity preparation procedures. The prepared cavities were randomly divided into four groups in terms of the composite resin type and its temperature before placing in the cavity (n=15). The mesial and distal cavities in each tooth were restored with one type of composite resin randomly with different temperatures.

Group 1: Filtek P60 composite resin at room temperature

Group 2: Filtek P60 composite resin after preheating up to 68°C

Group 3: X-tra fil composite resin at room temperature 

Group 4: X-tra fil composite resin after preheating up to 68°C

A metallic matrix band in a Tofflemire matrix retainer was fixed around the teeth to create a uniform clinical condition.

Clearfil Liner Bond (Kuraray, Japan) adhesive system was used in all the cavities according to the manufacturer’s instructions before placing composite resins in the cavities. First, the self-etch primer was applied to the cavity walls for 20 seconds and spread on the walls with an air syringe. Then the bonding agent was applied to all the cavity walls and homogeneously spread on the walls and light-cured for 10 seconds with an LED light-curing unit (Bludent LED smart, Bulgaria) at a light intensity of 1300 mW/cm2, with the light-conducting nozzle perpendicular to and barely touching the occlusal margins.

In group 1, the cavities were restored with Filtek P60 composite resin at room temperature (23°C) in two 2-mm layers and each layer was light-cured for 20 s.

In group 2, first, the Filtek P60 composite resin was heated up to 68°C in a Calset unit (Ad Dent Inc, Danbury, CT, USA) and placed in cavities in two 2-mm layers. In order to decrease heat loss during the placement of each layer, the maximum time for retrieving the composite resin from the heating unit and placing it in the cavity was 10 seconds. After adapting each layer of composite resin and 15 minutes of cooling (to decrease thermal shrinkage), it was light-cured for 20 seconds (11).

In group 3, X-tra fil composite resin was placed in the cavity in bulk at room temperature in a 4-mm layer and light-cured for 20 seconds.

In group 4, X-tra fil composite resin was preheated up to 68°C, using the same technique as that in group 2. Then it was placed in bulk in the cavity immediately and allowed to cool for 15 seconds, followed by light-curing for 20 seconds.

A thin layer of nail varnish was used on the occlusal surfaces in each group with different colors for identification. All the restorations were finished and polished with aluminum oxide disks from coarse to fine (Soft-Lex, Tin, 3M, ESPE USA) according to manufacturer’s instructions and incubated in distilled water at 37°C for 24 hours. A 500-round thermocycling procedure was carried out at 5°C/55°C, with a dwell time of 30 seconds and a transfer time of 10 seconds in a water bath (12). Then the roots of all the teeth were removed at a point apical to the gingival margins of restorations, and the crowns were sectioned into mesial and distal halves with a diamond disk (Resista, Italy).

To evaluate the marginal gaps of the samples under an electron microscope, first, the samples were fixed on the aluminum tabs and evaluated under an SEM (VEGA II TESCAN, Czech) after drying, preparation and gold-spattering. After visualizing the limits of the restoration at various magnifications, the maximum width of the marginal gap of the restorations was measured at ×2000 magnification in µm at buccal, lingual (enamel margins) and gingival (dentin margins). The width of the gap was determined in µm by placing two points on each side of the gap (one on the composite resin side and the other on the tooth side) and measuring this distance with a software program.

Data were analyzed with SPSS 21. One-way ANOVA was used to compare the mean marginal gaps at enamel and dentin margins between the four study groups. Post hoc Tukey tests were used for two-by-two comparisons of the groups. Paired t-test was used to compare the gaps at enamel margins with those at dentin margins in each group. Statistical significance was set at *P*<0.05.

## Results

Table 2 presents the means and standard deviations of restoration margin gaps at enamel and dentin walls with Filtek P60 and X-tra fil composite resins at the evaluated thermal conditions.

**Table 2:**
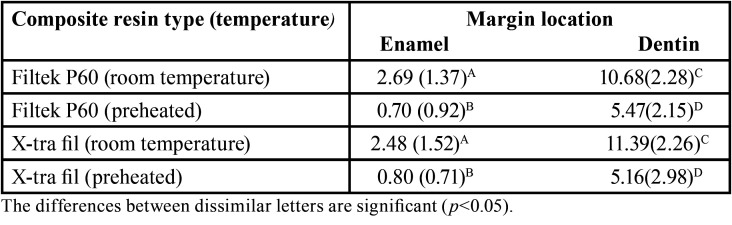
The means (standard deviations) of marginal gap measurements in study groups in µm.

One-way ANOVA showed significant differences in gap formation between the four study groups at the enamel wall– and dentinal wall–composite interfaces (*P*<0.0001).

Two-by-two comparisons of the groups with post hoc Tukey tests showed lower mean marginal gaps at both enamel and dentin margins with both composite resins with the use of the preheating technique (*P*<0.0001). However, comparison of the gaps at both enamel and dentin margins between the two composite resin types did not reveal any significant differences in room temperature and preheating conditions at enamel (*P*=0.96, *P*=0.99 respectively) and dentin margins (*P*=0.85, *P*=0.98 respectively).

**Figure 1:**
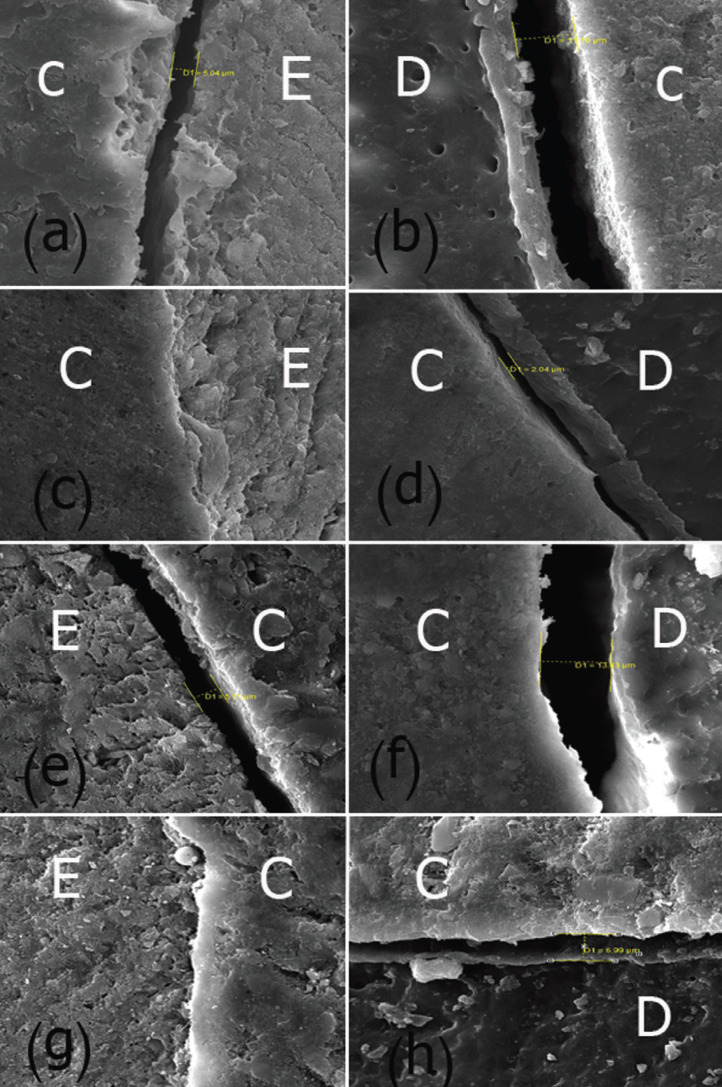
Evaluation of gap formation under a scanning electron microscope at ×2000: group 1 (a,b), group 2 (c,d) , group 3 (e,f) and group 4 (g,h) at enamel/dentin-composite interface. C: composite resin; E: enamel; D: dentin.

Based on the results of paired t-test, in all the study groups, the mean marginal gaps at the enamel walls of the restoration were significantly less than those at the dentin walls (*P*<0.0001), (Fig. 1).

## Discussion

The present study investigated the effect of preheating two types of packable composite resins (Filtek P60 and X-tra fil) on the marginal adaptation on the enamel and dentin walls of Cl II restorations. Based on the results, the mean marginal gaps on the enamel and dentin walls with the use of both types of composite resin after preheating up to 68°C were significantly lower compared to cavities restored with composite resin at room temperature. In this context, Fores-Salgado *et al* (13) and Elsayad (14) reported better adaptation and lower marginal gaps with preheating of composite resins. In a study by Alizade Oskoee *et al*, the formation of gaps at the gingival margins of Cl V cavities decreased with the preheating technique (15).

Since composite resin is a viscoelastic material, an increase in temperature decreases its viscosity and increases its liquidity (16), which is due to the thermal vibration of the resin monomers and an increase in their separation. Under these conditions, if the film thickness of the resin decreases and if it is placed in the cavity rapidly, it is easily adapted with the cavity walls (17). Therefore, a decrease in the marginal gaps after preheating the composite resin can be justified. However, an increase in the temperature and the motility of the radicals and monomers might affect the degree of conversion of the composite resin (18). An increase in the conversion rate increases the polymerization shrinkage and shrinkage stresses (19). Based on these reasons, Lohbaur *et al* reported the detrimental effects of preheating on the margins of composite resin restorations (20). In fact, polymerization shrinkage in association with thermal contraction affected the marginal seal through the creation of stresses at preheated composite resin–tooth interface. Elhejazi *et al* suggested a delay of 15 seconds after carrying the preheated composite resin into the cavity and before curing it to solve such a problem (11). A delay in curing the preheated composite resin decreases the temperature so that the conversion rate does not increase; however, the temperature is it still adequately high to allow proper wetting of the cavity walls (17). Under the clinical conditions similar to that in the present study, when the composite resin is heated after its rapid transfer to the cavity, there is an interval between shaping and curing. In addition, some studies have reported no differences in the marginal adaptation of composite resin restorations with the preheating technique (21,22). Differences in the behavior of materials in response to heat and differences in their initial viscosity might explain the disparities in the results of studies. Blalock *et al* (6) showed no relationship between the flow and composite resin type, filler content and the shape of the particles.

Another finding of the present study was a lack of difference in the efficacy of these two microhybrid composite resins (Filtek P60 and X-tra fil) and the formation of gaps at enamel and dentin margins. The structure of composite resin and the mechanism of its placement in the cavity are two factors that affect the marginal adaptation of restorations. Contrary to conventional composite resins (Filtek P60) which require incremental placement in the cavity to ensure proper penetration of light and adequate polymerization (23), bulk-fill composite resins (X-tra fil) can be placed in 4–5-mm thicknesses. In these composite resins, use of polymerization modulators technology has resulted in higher flexibility during polymerization and deeper penetration in the layers placed in bulk in the cavity (9).

Consistent with our results, in a study by Behery *et al* (24), gingival margin microleakage of Cl II cavities restored with bulk-fill composite resins (Tetric Evoceram bulk fill, X-tra fil, QuiXX) was not different from that of conventional composite resins (TPH Spectra HV). The disparities between the results of different studies might be attributed to the higher flexibility of bulk-fill composite resins, which affects decreases in shrinkage stresses of the composite resins placed in bulk.

Another finding of the present study was the lower marginal gaps at enamel margins compared to dentin margins in all the study groups, consistent with the majority of previous studies (25,26). The homogeneous structure of the enamel results in a more reliable bonding with it, while it is more difficult to achieve a proper bond with dentin, which might be attributed to its lack of homogeneity, the flow of tubular fluids to the cavity surface and its lower mineral content compared to enamel (27). When the stresses resulting from polymerization shrinkage are higher than the initial bond strength of composite resin to dentin, gaps are formed at composite resin–dentin interface (28).

In general, it might be claimed that a decrease in viscosity and an increase in the wetting property of composite resins due to heat might decrease gap formation in composite resin restorations. In addition, use of bulk-fill composite resins (X-tra fil) in posterior teeth, with advantages such as shorter placement time in bulk and performance similar to that of conventional composite resins (Filtek P60), might be useful if complete polymerization is carried out. However, further studies with conditions more similar to clinical situations with the use of different composite resins are necessary.

## Conclusions

Under the limitations of the present study, it can be concluded that:

1. The gaps at enamel and dentin margins of Cl II cavities decreased when both Filtek P60 and X-tra fil composite resins were applied after preheating.

2. Both composite resins evaluated exhibited similar effects on marginal gap formation.

3. There were less marginal gaps on enamel walls compared to that on dentin walls.
